# A collection of public transport network data sets for 25 cities

**DOI:** 10.1038/sdata.2018.89

**Published:** 2018-05-15

**Authors:** Rainer Kujala, Christoffer Weckström, Richard K. Darst, Miloš N Mladenović, Jari Saramäki

**Affiliations:** 1 Department of Computer Science, Aalto University, P.O. Box 15400, FI-00076 Aalto/Espoo, Finland; 2 Department of Built Environment, Aalto University, P.O. Box 14100, FI-0076 Aalto/Espoo, Finland

**Keywords:** Geography, Computational science, Databases, Civil engineering, Scientific data

## Abstract

Various public transport (PT) agencies publish their route and timetable information with the General Transit Feed Specification (GTFS) as the standard open format. Timetable data are commonly used for PT passenger routing. They can also be used for studying the structure and organization of PT networks, as well as the accessibility and the level of service these networks provide. However, using raw GTFS data is challenging as researchers need to understand the details of the GTFS data format, make sure that the data contain all relevant modes of public transport, and have no errors. To lower the barrier for using GTFS data in research, we publish a curated collection of 25 cities' public transport networks in multiple easy-to-use formats including network edge lists, temporal network event lists, SQLite databases, GeoJSON files, and the GTFS data format. This collection promotes the study of how PT is organized across the globe, and also provides a testbed for developing tools for PT network analysis and PT routing algorithms.

## Background & Summary

Public transport (PT) networks help to provide efficient and sustainable mobility in cities around the world^1^. PT network structure and schedules are challenging to plan, as one has to specify both the static structure of the network as well as the detailed schedules of PT vehicle departures. In particular, planners should have adequate tools for the analysis and optimization of the PT networks^2–6^, as well as expertise on how PT networks are structurally organized across cities^7–12^. For developing such tools and quantifying features of efficient public transport systems in different cities, detailed data on PT network operations are required.

Fortunately, an increasing number of public transport agencies publish their route and schedule data with the General Transit Feed Specification (GTFS, https://developers.google.com/transit/gtfs/) as the standard, open format. GTFS specifies how to present PT service supply with a series of CSV (comma-separated-values) plain text files constituting a GTFS *feed*. GTFS data is primarily used for PT passenger routing, but it can also be used for research, for instance for modeling PT-provided accessibility^4,6,13^.

GTFS data are increasingly available through the web sites of PT agencies as well as online repositories such as Transitland (http://transit.land) and TransitFeeds (http://transitfeeds.com). However, using GTFS data for studying how PT is organized in different cities remains challenging. The first challenge is that the PT timetable data covering a city are often fragmented into multiple feeds, each describing the operations of a single PT operator. These multiple feeds must be merged to cover all relevant modes of transport in the area of interest. The second challenge is the opposite of fragmentation: sometimes feeds are provided for large areas, such as whole countries, and city-level data has to be filtered from the feeds spatially, which requires specifying city boundaries. The third challenge is that GTFS data may contain errors, e.g., in the coordinates of PT stops, or times of operations. Thus, the data have to go through a set of validation steps before they are used. The fourth and final challenge is that learning the details of the GTFS standard can be time-consuming and slow down the adoption of GTFS data; network scientists, for example, are much more familiar with other data formats.

**Figure 1:**
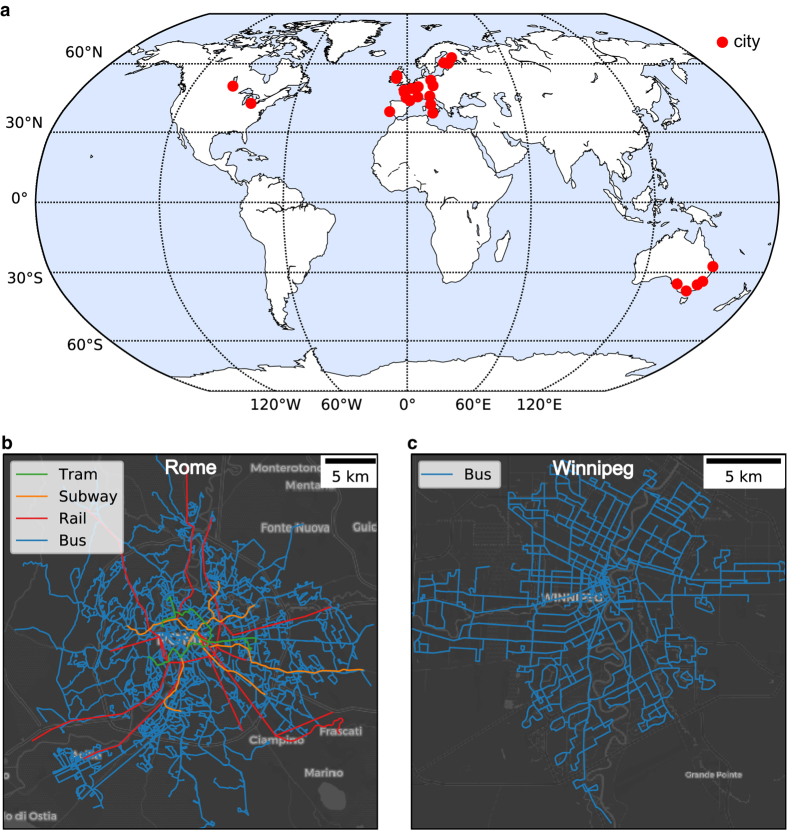
Spatial overview of the included cities, and two example PT network layouts. In panel **a**, we show the spatial overview of the cities' locations included in the collection. In panels **b** and **c**, we show two example PT network layouts for the cities of Rome and Winnipeg. In Rome the PT network covers four different modes of travel (tram, subway, train, and bus), while the PT network of Winnipeg is operated solely based on buses. Background map courtesy of OpenStreetMap contributors, and Carto. Rome route network data used for visualization is published by Roma servizi per la Mobilità under CC BY 3.0 IT (https://creativecommons.org/licenses/by/3.0/it).

The above challenges call for easy-to-use, validated data sets on urban public transport networks. However, to the best of the authors' knowledge, the only such data set is the multi-modal, temporal public transport network of Great Britain^14^. To this end, we have published a collection of PT network data sets for 25 cities in multiple, easy-to-use data formats. The locations of these cities, as well as examples of two computer-generated route maps are shown in Fig. 1. The provided formats include SQLite databases, GeoJSON-files usable for GIS-based analysis, static network edge lists and temporal network event lists commonly used by the network scientists, as well as the GTFS data format.

To compile this collection of PT networks, we first downloaded the relevant GTFS feeds for each of the 25 cities, and then imported these feeds into a database, sometimes merging feeds from multiple sources. Then, we checked the data for errors, and filtered the databases to match the spatial bounds of the cities. As GTFS feeds do not always contain information on transfer times between stops, we augmented the GTFS data with stop-to-stop walking distances using street network data from the OpenStreetMap project (http://www.openstreetmap.org, https://planet.osm.org).

The primary aim of publishing this collection of extracts is to facilitate comparative research on PT networks. However, the collection can be used for other purposes too. For instance, the city extracts can be used as a test bed for developing computational methods for PT network analysis and routing algorithms^15^. To make the collection of PT data sets easy to access, we have also launched an on-line repository http://transportnetworks.cs.aalto.fi that allows interactive and visual exploration of the data.

## Methods

For background, we introduce the structure and contents of a GTFS feed by listing the files in the feed and their contents in Table 1. As the different modes of PT transport, such as “bus” or “tram”, are relevant in our data processing pipeline we list also their definitions, numeric codes, and short tag names in Table 2. For more detailed information on the structure of each file and how they relate to each other, please see https://developers.google.com/transit/gtfs/reference/.

**Figure 2:**
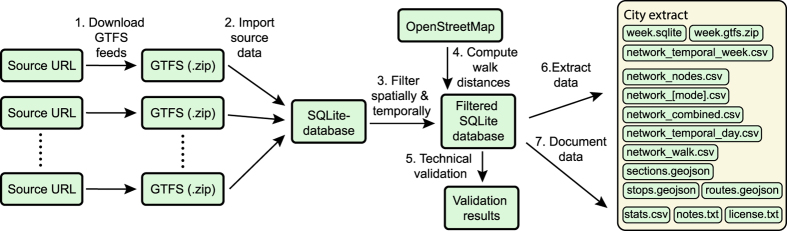
Data processing pipeline for one city. The pipeline for data processing consist of multiple subtasks. In the figure, the numbers indicate the order in which different tasks are carried out. Please see the main text for more details on each step and the final data formats.

Our data processing pipeline for producing the city extracts consists of multiple steps, as shown in Fig. 2. Each of these steps is detailed below.

### Step 1: Selecting cities and downloading source data

When selecting the cities whose PT networks are to be included in our collection, we focused on covering cities of different sizes, from different continents, and from various geographies. The final selection of cities was, however, heavily affected by the availability of data, as many cities do not yet publish their data in the GTFS format. In addition, the licensing terms for the source data affected our selection of cities. Many cities and countries that publish GTFS data provide non-standard custom licenses that can be hard and time-consuming to interpret, licenses that do not allow modification or redistribution of the data, or no licensing information at all. Thus, for practical reasons and to guarantee free data reuse for scientific purposes, we have included only cities for which there was data available under public domain or one of the commonly used open data licenses, such as the Creative Commons -licenses or the Open Database License (ODbL) by the Open Data Commons. All these licenses allow redistributing the data and using it for scientific purposes. The exact licensing terms for the data of each city are provided alongside each data extract. The final list of the 25 selected cities is provided in Table 3 and these cities are visualized on the world map in Fig. 1.

In the process of discovering the GTFS data for each city, we searched the websites of PT agencies as well as known GTFS data repositories for the download URLs of the GTFS feeds. The data was then downloaded by Python scripts developed in-house that take care of possible authentication issues, store the downloaded data in a consistent manner on our premises, and allow for continuous re-downloading of the data on a weekly basis.

### Step 2: Importing data into SQLite databases

In the second step, for each city, we imported the GTFS data into a relational SQLite database. An SQLite database is a single file that consists internally of different *tables*, each having their own sets of columns and rows. Due to the optimized structure and indexing, an SQLite database can be efficiently queried using Structured Query Language (SQL).

The table structure of the created SQLite database matches closely with the original tabular format of the data. Typically, each of the .txt files was imported into a table having the same name as the original file without the .txt extension. Additionally, to enable fast querying of the data, we created two convenience tables: table days enables querying the trips that run on each day, and table day_trips2 enables fast discovery of trips that take place between two arbitrary time points expressed in Unix time. Furthermore, we created a new table, stop_distances, which lists the distances between stops that are at most 1,000 meters apart from each other, when measured using great-circle distance. Last, we added a metadata table for storing auxiliary data on the feed as well as some pre-computed statistics.

When importing the data into a database, we changed any extended GTFS route types (describing the mode of travel) (https://developers.google.com/transit/gtfs/reference/extended-route-types) into the standard set of route types (tram, subway, rail, bus, ferry, cable car, gondola, funicular) specified by the GTFS. This transformation was done in order to make comparisons across cities easier.

For faster querying of the data and storage efficiency, we replaced the original identifier columns containing strings (*_id) with columns (*_I) having integer keys in all tables, except for the identifiers (shape_id) of the shapes.txt file. For instance, after the transformation, the main string identifier of each stop, stop_id, was replaced by an integer-valued column stop_I. However, the original string identifiers (*_id) were also retained in those tables where the _id field is used as the primary key mapping a row into a unique entity. Thus, for instance, in the stops table (describing the details of each PT stop) we preserved the stop_id column, but not in the stop_times table, as this data is excessively redundant.

Typically, the schedules for each PT route are specified by listing the stop times for each run of a route in the stop_times.txt file. However, sometimes the operations on a route are specified through the frequencies.txt file, providing the headway of a route but no departure times at the departure stop. In this case, the travel durations between consecutive stops are nonetheless specified in the stop_times.txt. To unify the structure of the data in the SQLite database and the data extracts, we expanded such frequency-encoded route operations into multiple regular trips (route runs), resulting in additional rows in the trips, days, day_trips2 and stop_times tables.

If a city's PT operations were described in multiple feeds, we merged the feeds into a single database while prefixing all *_id columns separately for each feed. Otherwise, the data importing process progresses as usual.

For some cities, there were multiple stops with identical geospatial coordinates in the original data. To remove such duplicated stops, we aggregated together all stops that were less than one meter apart from each other. To do this, we first grouped the stops so that all stops that are less than one meter apart belong to the same group. Then, for each group, we selected one of the group's stops to represent all stops in the group. Finally, to remove the duplicated stops, the information on the other, non-representative stops were removed from stops and stop_distances tables, and their entries in stop_times were updated to use the representative stop's stop_I identifier.

While the SQLite imports are basically a direct import of the input CSV files, a researcher already benefits from them for multiple reasons. First, the import step converts semi-structured GTFS data into highly structured tabular data and catches any obvious inconsistencies. Second, different methods of representing the same thing (e.g. exact stop times vs. frequencies) are converted to a single, consistent representation. Third, SQLite is a common, open source format which can be used for ad hoc querying and data exploration much more easily than the original text files.

### Step 3: Spatial and temporal filtering of the data

To ensure ease-of-access and consistency across the provided data extracts, we filter the SQLite databases both in space and time. The spatial filtering of the data is required for removing long-distance PT connections reaching far out from each city's urban area. Temporal filtering is performed for two reasons. First, we aim to cover typical operations, without any irregularities such as local public holidays that take place on working days. Second, given that PT time tables are commonly scheduled on a weekly basis, we select a representative one-week time period that should fully capture the provided PT services in a city, as PT services are commonly planned to be week-periodic and variations in PT services across neighboring weeks are typically small. This also ensures consistency and comparability of results in future use of the data set, as the time-period of the data extracts are precisely defined. The final parameters used for spatial and temporal filtering of the data are provided in Table 4.

### Spatial filtering

Spatial filtering of a GTFS feed is challenging for two reasons. First, the non-administrative areal limits of a city or metropolitan area can be difficult to define, and multiple different definitions exist^16^. Second, PT routes can span large areas, which needs to be taken into account when filtering the data.

While one could argue for different definitions of a city area, most likely no spatial filtering approach is ideal for all use cases. In our spatial filtering approach, we decided to pursue these goals: (i) limit the spatial extent of the city extracts to the approximate spatial bounds of the city, (ii) include only PT connections within the spatial bounds of the city, and (iii) cause minimal artifacts in the resulting data extracts, and the PT network structure, due to cutting of PT trips.

To this end, we adopted the following approach. First, we defined the extents of each city heuristically based on the stop locations and on satellite imagery. Note that while we could have also used administrative boundaries of the city, these may not properly reflect how the PT network is planned. For each city, we then selected a central point and a radius around this central point that should cover all the continuous and dense parts of the city and its PT network. The central point was selected to be approximately at the center of the city area, if the shape of the urban city area was approximately round. If the city area was very asymmetric, the central point was chosen to be at the city center, e.g. close to to the main railway center. Then we preserved all PT operations residing within this buffer area.

**Figure 3:**
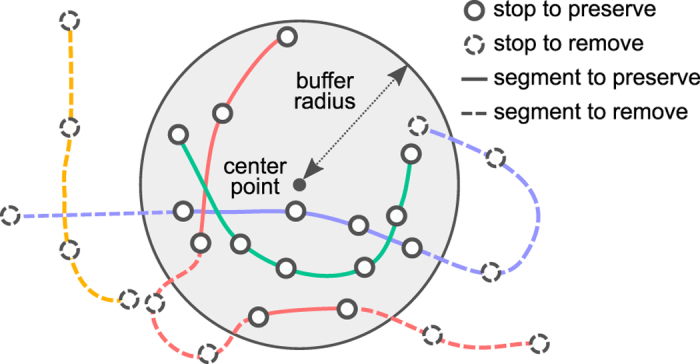
Spatial filtering of a GTFS feed based on stop coordinates. The city area is defined by a center point and an associated buffer radius. Only PT connections within the defined city area are included. Each line and color corresponds to a different PT trip (=one run of a PT route). When necessary, a trip is split into multiple parts so that only those parts of the trip are retained which run within the defined city area. Here, the red PT trip is split into two parts, as it temporarily exits and then re-enters the defined city area.

**Figure 4:**
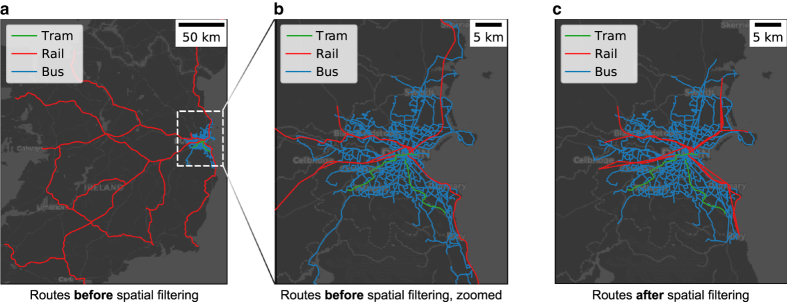
The importance of spatial filtering. The figure shows the PT routes of Dublin's GTFS source data before and after the spatial filtering. In panels **a** and **b**, we can see that the train routes present in one of the source feeds extend well beyond the city borders of Dublin. In panel **c**, we show the situation after spatial filtering, where the non-urban parts of the railway and bus networks have been removed. Note that, there are also some minor changes in the routes that reside within the buffer radius used for Dublin (20 km). These differences are not present in the actual data but are due to the visualization approach, which shows for each PT route the spatial shape of only one run of the route for efficiency reasons. Notably, the Dublin Area Rapid Transit (DART) route splits into two branches northeast from the city center. Due to the chosen visualization approach, this is now seen as a small discrepancy in the rail networks extending east from the city center, although this discrepancy is not present in the actual data.

If a PT trip (=one run of a PT route) temporarily exited the buffer area, but later returned back, we split the trip into two or more subtrips, with each subtrip going through only stops that reside within the defined city area. In this case, the original trip is replaced in the SQLite database by the split trips, which were assigned a trip_id of the form [original_trip_id]_splitted_part_{i}, where {i} stands for the ordinal number of the split trip. We chose to split trips that go outside the defined city area in order to prevent artifactual PT connections, which would have been emerged, e.g., if we would have only removed the stop time entries occurring outside the defined city area. We illustrate the spatial filtering approach in Fig. 3. An example where spatial filtering was especially necessary is showcased in Fig. 4

All trips that had only one entry in the stop_times table were removed from the trips table along with the corresponding entries in the stop_times table. Finally, all other tables' rows which are no longer “referred to” by any other tables are removed from the SQLite database.

### Temporal filtering

**Figure 5:**
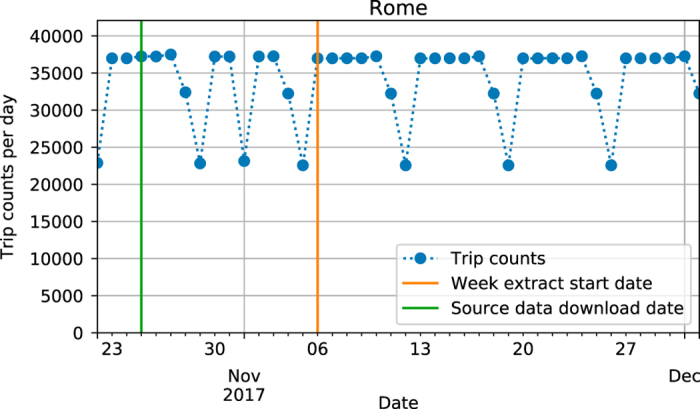
Temporal filtering is required for selecting a representative week and day. The daily variations in PT trip counts for Rome show the regular weekly pattern in the provided GTFS data. On 1st of November, the All Saints Day, we can see a drop in PT services due to the public holiday. Because of this, the next full week after All Saints' Day is selected as the representative week.

The dates for the temporal filtering were chosen based on the number of daily trips of the included feeds. We ensured that there was a clear weekly periodicity in the feeds, so that working days have roughly the same number of trips and that weekends have a different schedule. Then we selected the dates for the week-long and day-long extracts, where we aimed for a typical work week without major exceptions in the PT operations, such as national holidays. To this end, we ensured that all weekdays have at least 90% of the maximum number of daily PT trips during the time span of the week-long extract. This enabled us to select the extract time spans in a semi-automatic manner, as illustrated in Fig. 5. If a city's timetable data consisted of multiple GTFS sources, we also ensured that each of the feeds was valid for the dates selected.

After the week- and day-extract start dates have been selected, our temporal filtering process preserves all PT trips in the GTFS data that depart within the precise timespan of the selected week or day. The precise time-span for the weekly extracts was set to range from 03:00:00 on Monday to 02:59:59 on the next Monday, and for the daily extracts from 03:00:00 on Monday 02:59:59 on the next day. We chose 3AM to be the “cutting point” as at this hour of day there were typically very few number of PT trip departures in all of the included cities. Note, that while the data contains no PT events before the beginning of the defined time-span, there can be PT events taking place after the end of the defined time-span, as some trips starting before the end of the time-span are still operating after the end of the time span.

In practice, the filtering process required removing entries from the calendar, calendar_dates, days, and day_trips2 tables, and adapting the service information in the calendar, calendar_dates and trips tables for the preserved trips. Finally, all other tables' rows which are no longer being “referred to” by any other tables are removed from the SQLite database.

### Step 4: Computing walking distances between stops

As GTFS data format does not require one to provide transfer times or walking distances between stops, we computed stop-to-stop distances for each city to enable accurate modeling of walking transfers between stops. To limit the number of stops pairs, we computed stop-to-stop distances only between stops that were less than 1 km apart from each other. To this end, we used data from OpenStreetMap covering the whole planet (https://planet.osm.org/, OpenStreetMap contributors). For the actual routing, we used the open source GraphHopper routing library https://github.com/graphhopper/graphhopper, and augmented the SQLite databases with these results. After the walk routing had been performed, all pairs of stops that were found unreachable by the OpenStreetMap-based pedestrian routing were removed from the stop_distances table. This would be the case e.g. when one of the two stops resided on an island, from which there is no bridge to the mainland, where the other stop resides. In few cases, where OpenStreetMap is lacking accurate data, the routing can cause PT stops to become unconnected in the walking network or the walking distance to become artificially large. However, the accuracy and coverage of OpenStreetMap data is typically so good that it can be used for reliable pedestrian and bike routing in public transport journey planners.

### Step 5: Data validation

At this stage, we performed validation steps for the SQLite database. Please see Section “Technical Validation” for more information.

### Step 6: Creating extracts from the city databases

As a final step of our pipeline, we extracted data from the database, and created the extracts described in Section “Data Records”.

### Code availability

Alongside the data, we share also our software and scripts for the full data processing pipeline. All code has been written for Python 3.5 and has been tested to work on both Mac and Linux. This code for downloading the original data and processing of the data into the city-sized extracts is available at https://github.com/CxAalto/gtfs_data_pipeline^17^. Internally, this pipeline heavily uses our in-house developed Python package gtfspy (https://github.com/CxAalto/gtfspy)^18^. The Java code for running the pedestrian routing is provided alongside the gtfspy Python package. Both software resources are available under the MIT license (https://opensource.org/licenses/MIT).

## Data Records

For each city, we provide the extracts listed in Table 5, which are then discussed in more detail. For long term archival, a bulk copy of all data is provided at Zenodo (Data Citation 1). In addition, we have also launched our own data repository http://transportnetworks.cs.aalto.fi that allows for interactive exploration and visualization of the data sets. Some of the provided data sets (week.sqlite, week.gtfs.zip, network_temporal_week.csv) cover the operations of the full week, while the rest cover operations during on typical working day (Monday).

### Week extracts

- week.sqlite: The week.sqlite database is our master SQLite database for each city. This database has pre-built indices that allow efficient querying of data for many use cases. For more details on the structure of the SQLite database, see the Section “Step 2: Importing data into SQLite databases” under Methods.
- gtfs.zip:
gtfs.zip is a GTFS export of the week.sqlite database covering the operations taking place during one week and has been spatially filtered to match the city bounds. Note that here any operations that were originally coded using the frequencies.txt file are expanded into the stop_times.txt files and thus no frequencies.txt file is present.
- network_temporal_week.csv: To allow easy investigation of the data using temporal network methodology^19^, we provide a week-long temporal network extract listing the elementary PT connections, or *events*, that describe the progression of a PT vehicle from a stop to its next stop along the route. Note that the rows in the file are not sorted by the departure time of the events, and that the values for trip_I repeat across the days. For more detailed description of the contents, see Table 6.

### Day extracts

The network and GeoJSON extracts are aggregations of one working day (Monday). The precise descriptions of the contents of each file are provided in Table 6, and Table 7 describes data type for each data field. Below, we outline the content of each of the extracts.

#### Network extracts

All data related to the network extracts are provided as comma-separated-values files (.CSV) where semicolon (";") is used as the delimiter. Below we list the contents of each file.

- network_nodes.csv:Information on the PT stops that function as the nodes of the network.
- network_walk.csv:This file describes the stop-to-stop walking distances between network nodes (stops). In the file we provide both euclidean (straight-line) distances and the values computed using Open Street Map routing. Note that links exist only between stop pairs where the straight-line distance d is smaller than 1000 meters, and there is a footpath connection between the stops.
- network_[mode].csv:To enable easy investigation on the role of different PT modes, we provide a file network_[mode].csv describing the operations for each mode of PT. Here, the [mode] part of the file name is one the values listed under the column “Mode tag” in Table 2. For instance, the tram network of a city would be provided in file network_tram.csv. If there are no operations for a mode of PT, then no network_[mode].csv file is provided.
- network_combined.csv:The file network_combined.csv contains the combined set of links of the mode-wise networks (network_[mode].csv). Note that there can be multiple links between two stops that correspond to different modes of travel, differentiated by the route_type field.
- network_temporal_day.csv:Similarly to the network_temporal_week.csv, we provide a listing of temporal network events for the specified Monday matching the other Monday-related data extracts. Note that the rows in network_temporal_day.csv are not sorted by the departure time of the events.

#### GeoJSON extracts

To enable the data to be used in popular GIS-software and web-mapping tools, we provide also GeoJSON files for the stops, sections (i.e. PT network links) between the stops, and routes. All coordinates in the GeoJSON files are expressed in the WGS84 coordinate system.

- stops.geojson:Information on the public transport stops.
- sections.geojson:All stop-to-stop sections with data on operations.
- routes.geojson:In this file we provide the stop coordinates for each route. Here, a distinct route is specified by the GTFS standard using the field route_id. Note that even if a route would operate in two directions, only one direction of the route is included. Also, the GTFS standard allows routes to go through different stops on different trips. Here, we provide the coordinates of stops of the trip that has the longest scheduled duration.

## Metadata

### license.txt

The licensing terms of each city extract are described in license.txt -files. The legal code of the referenced licenses are provided in an additional file [x]_legalcode.txt, where [x] stands for an abbreviation of the license name under which the data is provided.

### notes.txt

In notes.txt we state how comprehensively the each city extract covers the PT services available within the city area. Sometimes we also provide minor additional remarks regarding the feed.

### stats.csv

The statistics file containing the following summarizing the operations taking place on a Monday. The details of the provided statistics are listed in Table 8.

## Technical Validation

Our validation process consists of two major steps: automated data validation through various sanity checks and comparison of the data to openly available information on the PT network structures. The first step of our automated data validation is to check that the importing of the GTFS files into SQLite databases has been successful and there are no missing data.

For detecting actual errors in the schedule data, we computed for each city the number of cases (in the week.sqlite database) where:

1. distance between consecutive PT stops on a trip is longer than 20 km;
2. five or more consecutive stops on a trip have the same scheduled arrival time;
3. trip duration is longer than 2 hours;
4. travel duration between consecutive stops is larger than 30 minutes;
5. the average speed of a PT trip is unrealistically high with respect to the PT mode in question. The mode-wise threshold speeds used for detecting unrealistically fast PT trips are listed in Table 2.

Most feeds raise warnings for at least some of these features. For instance, it is typical that five or more consecutive stops along a trip have been sloppily scheduled to have same arrival time, when the PT vehicle is heading towards its depot. Sometimes, on the other hand, these warnings are simply false alarms. Regardless whether such warnings are due to errors in the source data or are just peculiarities of the city's PT operations, researchers using the data should be aware of them. Therefore, we report the numbers of the above-issued, computer-generated warnings in file week_db_timetable_warnings_summary.log.

After the creation of the data extracts, we perform a visual sanity check of the data for each city. At this stage, we visualize all PT routes of each city on top of a map using an associated web-mapping tool (https://github.com/CxAalto/gtfspy-webviz) and assess whether the locations of the PT routes seem viable with respect to the surrounding land use, and that the spatial filtering process has been successful. In addition, we use the web-mapping tool to animate the movement of PT vehicles in time, and check that there are no striking peculiarities in the data, such as a PT vehicles suddenly jumping between distant locations.

**Figure 6:**
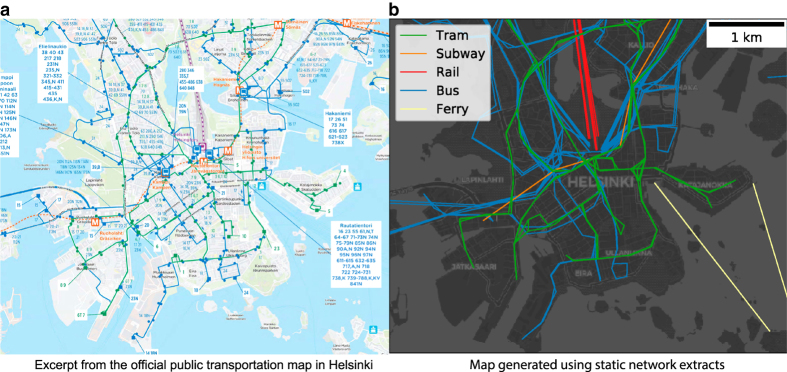
Validating the coverage of the data extracts using publicly available PT route maps. In the figure, we compare the official route map of Helsinki to a computer-generated map created based on the static network extract for Helsinki. In panel **a**, we show an excerpt of the official PT transportation map of Helsinki city center published in early 2018 (routes and layout: Helsinki Region Traffic, background map OpenStreetMap). The blue, green, orange, and purple lines correspond to bus, tram, subway, and train routes, respectively. In panel **b**, we show the computer-generated map used for validation purposes (background map OpenStreetMap). Note that on the computer-generated map, the lines do not follow the actual paths taken by the PT vehicles, but connect the two stops directly. By comparing the two maps, we can see that both maps contain same modes of PT and most PT lines can be found in both maps. However, given that the maps cover slightly different time frames, there are also some differences in the maps due to changes in the PT network. One such example is the new continuation of the subway line east from the city center.

Finally, we compare the computer-generated PT network visualizations to the publicly accessible information about the PT network and operations provided in each city, such as network maps and timetables. This process is illustrated in Fig. 6 where the official PT route map of Helsinki and a computer-generated route map based on the static PT network extracts are compared. Out of the 25 cities, we found all relevant urban public transport modes for 14 cities (Adelaide, Berlin, Brisbane, Canberra, Dublin, Helsinki, Kuopio, Luxembourg, Melbourne, Paris, Rome, Turku, Sydney, Winnipeg). For 9 cities (Belfast, Bordeaux, Grenoble, Nantes, Palermo, Prague, Rennes, Toulouse, Venice) only commuter trains or regional buses are missing from the data. For Detroit and Lisbon, services in a part of the city area are missing. For Lisbon data on the tram network is also missing. We report the above-listed cases in the notes.txt -file provided alongside each city extract.

## Usage Notes

The spatially and temporally filtered GTFS data can be analyzed using many pieces of software. Of these, we mention our in-house-developed Python package gtfspy which was the core software block for creating these data extracts, and is interoperable with the provided week.sqlite databases. The package provides methods e.g. for computing various PT network statistics, as well as performing routing and accessibility analyses on the network.

The GeoJSON extracts can be easily analyzed and visualized using GIS-software such as QuantumGIS, as well as various web mapping tools.

Regarding the network data formats, there are multiple network analysis libraries available, such as the popular networkx library (http://networkx.github.io/) that can be directly used for analyzing the data. Note that when using these data for network analyses, the nodes (PT stops) have been minimally spatially aggregated. Thus, e.g. large bus or metro stations with multiple platforms often result in multiple network nodes, which should be taken into account especially when performing static network analyses.

## Additional information

**How to cite this article:** Kujala R. *et al.* A collection of public transport network data sets for 25 cities. *Sci. Data* 5:180089 doi: 10.1038/sdata.2018.89 (2018).

**Publisher’s note:** Springer Nature remains neutral with regard to jurisdictional claims in published maps and institutional affiliations.

## Supplementary Material



## Figures and Tables

**Table 1:** The contents of a GTFS feed.

**Filename**	**Required**	**Defines**
agency.txt	Required	One or more transit agencies that provide the data in this feed.
stops.txt	Required	Individual locations where vehicles pick up or drop off passengers.
routes.txt	Required	Transit routes. A route is a group of trips that are displayed to riders as a single service.
trips.txt	Required	Trips for each route. A trip is a sequence of two or more stops that occurs at specific time.
stop_times.txt	Required	Times that a vehicle arrives at and departs from individual stops for each trip.
calendar.txt	Required	Dates for service IDs using a weekly schedule. Specify when service starts and ends, as well as days of the week where service is available.
calendar_dates.txt	Optional	Exceptions for the service IDs defined in the calendar.txt file. If calendar_dates.txt includes ALL dates of service, this file may be specified instead of calendar.txt.
fare_attributes.txt	Optional	Fare information for a transit organization's routes.
fare_rules.txt	Optional	Rules for applying fare information for a transit organization's routes.
shapes.txt	Optional	Rules for drawing lines on a map to represent a transit organization's routes.
frequencies.txt	Optional	Headway (time between trips) for routes with variable frequency of service.
transfers.txt	Optional	Rules for making connections at transfer points between routes.
feed_info.txt	Optional	Additional information about the feed itself, including publisher, version, and expiration information.
Despite the .txt filename extensions, all files are comma-separated-values (CSV) files. This table is an excerpt from the “General Transit Feed Specification Reference” (https://developers.google.com/transit/gtfs/reference/) by Google LLC, licensed under http://creativecommons.org/licenses/by/3.0/CC BY 3.0.		

**Table 2:** PT travel modes as defined by the GTFS standard.

**Mode tag**	**route_type**	**Description**	**Max. speed (km/h)**
tram	0	Tram, Streetcar, Light rail. Any light rail or street level system within a metropolitan area.	100
subway	1	Subway, Metro. Any underground rail system within a metropolitan area.	150
rail	2	Rail. Used for intercity or long-distance travel.	300
bus	3	Bus. Used for short- and long-distance bus routes.	100
ferry	4	Ferry. Used for short- and long-distance boat service.	80
cablecar	5	Cable car. Used for street-level cable cars where the cable runs beneath the car.	50
gondola	6	Gondola, Suspended cable car. Typically used for aerial cable cars where the car is suspended from the cable.	50
funicular	7	Funicular. Any rail system designed for steep inclines.	50
The data for “route_type” and “Description” columns are copied from the “General Transit Feed Specification Reference” (https://developers.google.com/transit/gtfs/reference/) by Google LLC, licensed under CC BY 3.0 (http://creativecommons.org/licenses/by/3.0). The column “Mode tag” tells the tag used for different PT modes when producing single-mode network extracts. The column “Max. speed” tells the speed limit that we use to flag PT trips, where the average speed of trip distances is unrealistic.			

**Table 3:** The cities included in the collection, and basic information about their PT network properties.

**City**	**Country**	**Stops**	**Links**	**Connections**	**License**
Adelaide	Australia	7 548	9 257	404 300	CC BY 4.0
Belfast	Northern Ireland	1 917	2 181	122 693	ODBL v1.0
Berlin	Germany	4 601	12 079	1 048 218	CC BY 3.0 DE
Bordeaux	France	3 435	4 039	236 595	ODBL v1.0
Brisbane	Australia	9 645	11 738	392 805	CC BY 3.0 AU
Canberra	Australia	2 764	3 218	124 305	CC BY 4.0
Detroit	USA	5 683	5 948	214 863	CC0
Dublin	Ireland	4 571	5 567	407 240	CC BY 4.0
Grenoble	France	1 547	1 682	114 492	ODBL v1.0
Helsinki	Finland	6 986	9 072	686 457	CC BY 4.0
Kuopio	Finland	549	704	32 122	CC BY 4.0
Lisbon	Portugal	7 073	8 982	526 179	CC0
Luxembourg	Luxembourg	1 367	3 234	186 752	CC0
Melbourne	Australia	19 493	21 737	1 098 227	CC BY 4.0
Nantes	France	2 353	2 779	196 421	ODBL v1.0 fr
Palermo	Italy	2 176	2 561	226 215	CC BY 4.0
Paris	France	11 950	14 781	1 823 872	ODBL v1.0 fr
Prague	Czechia	5 147	6 754	670 423	CC0
Rennes	France	1 407	1 671	109 075	ODBL v1.0
Rome	Italy	7 869	10 206	1 051 211	CC BY 3.0 IT
Sydney	Australia	24 063	28 815	1 265 135	CC BY 4.0
Toulouse	France	3 329	3 793	224 516	ODBL v1.0
Turku	Finland	1 850	2 341	133 512	CC BY 4.0
Venice	Italy	1 874	2 737	118 519	CC BY 3.0 IT
Winnipeg	Canada	5 079	5 846	333 882	PDDL
The columns Stops, Links, and Connections indicate the number of each entity in the daily PT network extracts. The number of “Connections” corresponds to the number elementary, time-dependent PT vehicle movements between consecutive stops of a trip during the time span of the daily extract.					

**Table 4:** Details of the spatial and temporal filtering parameters for each city.

**City**	**Latitude**	**Longitude**	**R (km)**	**Download date**	**Extract date**
Adelaide	−34.9213	138.5775	40	2016-12-07	2016-12-12
Belfast	54.6001	−5.9304	30	2017-10-30	2016-09-05
Berlin	52.5190	13.4029	30	2016-12-07	2016-04-25
Bordeaux	44.8412	−0.5751	30	2016-12-07	2016-12-12
Brisbane	−27.4580	153.0226	40	2016-12-07	2016-12-12
Canberra	−35.2767	149.1254	30	2016-12-14	2017-01-09
Detroit	42.3700	−83.0807	30	2016-12-07	2016-12-12
Dublin	53.3497	−6.2566	20	2016-12-07	2016-12-12
Grenoble	45.1772	5.7228	20	2016-12-07	2016-11-14
Helsinki	60.1733	24.9409	30	2016-12-07	2016-12-12
Kuopio	62.8945	27.6807	10	2016-12-07	2016-12-12
Lisbon	38.7096	−9.1420	30	2017-01-30	2016-11-21
Luxembourg	49.6111	6.1329	20	2016-12-07	2016-11-28
Melbourne	−37.8493	145.0793	50	2016-12-07	2016-12-12
Nantes	47.2133	−1.5516	20	2016-12-07	2016-12-12
Palermo	38.1186	13.3598	20	2016-12-07	2014-09-22
Paris	48.8619	2.3519	35	2016-12-07	2016-12-12
Prague	50.0846	14.4311	30	2016-12-07	2016-12-12
Rennes	48.1079	−1.6749	20	2016-12-07	2016-12-19
Rome	41.8963	12.4853	20	2017-10-25	2017-11-06
Sydney	−33.8269	151.0643	50	2016-12-14	2016-12-19
Toulouse	43.6021	1.4428	20	2016-12-07	2016-12-12
Turku	60.4491	22.2671	10	2016-12-07	2016-12-12
Venice	45.4882	12.2416	20	2016-12-07	2016-12-12
Winnipeg	49.8819	−97.1352	30	2016-12-07	2016-12-12
Columns “Latitude” and “Longitude” indicate the location of the defined city center point, and *R* is the buffer radius used for spatial filtering.					

**Table 5:** Data provided for each city.

**File**	**Description**
week.sqlite	An SQLite database covering the operations for a week.
week.gtfs.zip	A spatially and temporally filtered GTFS feed covering the operations taking place during a week.
network_temporal_week.csv	Describes the PT operations on the level of elementary connections taking place during one full week.
network_nodes.csv	Information on the nodes for the network extracts.
network_[mode].csv	Static networks for each PT mode specified by the GTFS standard. Includes statistics for each link computed based on the operations on a Monday.
network_combined.csv	A combined static network of all PT modes. Includes statistics for each link computed based on the operations taking place on a Monday.
network_walk.csv	A combined static network of all PT modes. Includes statistics for each link computed based on the operations taking place on a Monday.
network_temporal_day.csv	Describes the PT operations on the level of elementary connections taking place on a Monday.
stops.geojson	Information on the nodes in GeoJSON format.
sections.geojson	Each stop-to-stop section in GeoJSON format.
routes.geojson	Public transport routes in GeoJSON format.
stats.csv	Simple statistics describing the PT operations on a Monday.
notes.txt	Additional information such as the feeds' download dates, original source URLs, and time zone. We describe here also specialties of the data set, such as missing modes of PT.
license.txt	License information for the feed.
[x]_legal_code.txt	Legal code for the provided data license.
See the main text for more detailed description of each file.	

**Table 6:** Information contained by network and GeoJSON extracts.

**Extract**	**stop_I**	**latitude**	**longitude**	**stop_name**	**from_stop_I**	**to_stop_I**	**n_vehicles**	**duration_avg**	**route_I_counts**	**route_type**	**d**	**d_walk**	**dep_time_ut**	**arr_time_ut**	**trip_I**	**route_I**	**route_name**
network_nodes.csv	✓	✓	✓	✓													
network_walk.csv					✓	✓					✓	✓					
network_[mode].csv					✓	✓	✓	✓	✓		✓						
network_combined.csv					✓	✓	✓	✓	✓	✓	✓						
network_temporal_day.csv					✓	✓							✓	✓	✓	✓	
network_temporal_week.csv					✓	✓							✓	✓	✓	✓	
stops.geojson	✓	✓	✓	✓													
sections.geojson		✓	✓	✓	✓	✓	✓	✓	✓	✓							
routes.geojson		✓	✓							✓						✓	✓

**Table 7:** Explanations for fields used in the network and GeoJSON extracts.

**Column**	**Data type**	**Explanation**
node_I	integer	The id used for a PT stop.
latitude	float	Latitude expressed in WGS 84 coordinate system.
longitude	float	Longitude expressed in the WGS 84 coordinate system.
stop_name	string	Name of the stop as presented to PT passengers as presented in the original GTFS data.
from_node_I	integer	From node's / stop's identifier.
to_node_I	integer	To node's / stop's identifier.
n_vehicles	integer	Number of PT vehicles that have traveled between two stops within a time interval.
duration_avg	integer	Travel time between stops averaged over all PT vehicles rounded to one second accuracy.
route_I_counts	list (string)	A list of route_I's and the number of times each route has operated between two stops. For the network extracts, this data is formatted as a string where each element is written as ``route_I:count'' and different routes are separated by a comma. An example value for this field is thus ``1:3,2:131,10:93''. For the GeoJSON extracts, we provide these values as part of the JSON object's attributes. Please note that the definition of a route varies across the cities provided, and that routes can have deviations from their main paths for instance when traveling to and from a depot.
d	integer	Straight-line distance between two stops expressed in meters.
d_walk	integer	Distance between two stops computed using OSM data expressed in meters.
dep_time_ut	integer	Departure time of an elementary PT connection in a temporal network expressed in Unix time (number of seconds after 1.1.1970 00:00:00 UTC).
arr_time_ut	integer	Arrival time of an elementary PT connection in a temporal network expressed in Unix time.
trip_I	integer	Identifier for a trip.
route_I	integer	Identifier for a route.
route_name	string	Name of a route as shown to passengers.

**Table 8:** Contents of a stats.csv file describing PT operation statistics for one day.

**Variable name**	**Description**
n_stops	The number of stops used at least once.
n_connections	Total number of elementary connections.
n_links	The total number of stop pairs between which at least one elementary PT connection takes place.
network_length_m	The sum of all links' (excluding walk) great circle distances, expressed in meters.
link_distance_avg_m	Average link distance: network_length / n_links.
vehicle_kilometers	Vehicle kilometers traveled.
buffer_center_lat	Latitude of the center point used for spatial filtering.
buffer_center_lon	Longitude of the center point used for spatial filtering.
buffer_radius_km	The radius of the buffer circle used for the spatial filtering.
extract_start_date	The starting date of the weekly extract, and the date used for the daily extracts.
